# Unraveling the mechanisms of health management needs among rural elderly in underdeveloped Chinese regions: a machine learning approach to predictive model building and factor analysis

**DOI:** 10.1186/s12877-026-07368-z

**Published:** 2026-03-28

**Authors:** Siting Yang, Wuyou Zhang, Yuan Pan, Rong Zheng, Haidong Xu, Pinghua Zhu

**Affiliations:** 1 School of Information and Management, Guangxi Medical University, Nanning, 530021 China; 2 Department of Science and Technology, Guangxi Medical University, Nanning, 530021 China; 3 School of Humanities and Social Sciences, Guangxi Medical University, No.22, Shuangyong Road, Qingxiu District, Nanning, 530021 China

**Keywords:** Elderly, Rural communities, Health management, Influencing factors, Machine Learning

## Abstract

**Background:**

Global population aging is accelerating, with China among the countries experiencing the fastest and largest aging populations. This study aims to analyze the current health management(HMN) needs of rural elderly in underdeveloped regions of China. Machine learning algorithms were employed to construct predictive models and identify key influencing factors, providing empirical evidence for the development of targeted health management strategies.

**Methods:**

From August 2023 to January 2024, a convenience sample of 641 rural community elderly aged ≥ 60 years across four prefecture-level cities in Guangxi was surveyed using questionnaires. Predictor variables were chosen using LASSO regression and Logistic regression. The predictive efficacy of three machine learning models - Logistic regression, Random Forest, and XGBoost - was methodically evaluated. Variable contributions were assessed using SHAP values, and model validity and practical applicability were validated through the nomogram, ROC curve,calibration curves, and decision curve analysis.

**Results:**

Health management needs among rural elderly in China’s underdeveloped regions were relatively low (43.84%). SHAP interpretability analysis identified five key factors influencing health management needs. By conducting SHAP explainability analysis, five crucial factors affecting the healthcare requirements of the elderly were pinpointed. XGB exhibited superior predictive accuracy in both the training and validation datasets, achieving AUC values of 0.783 and 0.723, respectively.Independent samples t-tests revealed six critical individual factors affecting health management needs. The study identified three primary health management needs: regular health monitoring and screening, enhanced health management education, and targeted chronic disease management services.

**Conclusion:**

The XGB prediction model constructed in this study effectively identifies health management needs among rural elderly in underdeveloped regions of China. It is recommended that relevant authorities optimize the allocation of rural community health service resources and implement targeted health interventions based on the high-demand population characteristics identified by the model to promote active aging.

**Supplementary Information:**

The online version contains supplementary material available at 10.1186/s12877-026-07368-z.

## Introduction

 As global demographic structures continue to shift, aging has become an irreversible trend. China ranks among the world’s fastest-aging nations, with the largest elderly population [1]. According to China’s Seventh National Population Census (2020), individuals aged 60 and above reached 260 million, accounting for 18.7% of the total population—significantly exceeding the United Nations’ 10% threshold for an aging society. In the context of the global phenomenon of severe ageing, the challenges encountered by underdeveloped regions are particularly acute. Historical geographical factors and delayed economic development have led to a situation in western China and its border areas characterised by insufficient total medical resources and uneven distribution. This has exacerbated rural depopulation, resulting in a growing regional disparity in the accessibility of medical services.From 2000 to 2020, the proportion of Guangxi’s population aged 65 and above increased from 7.76% to 15.2% [2]. Guangxi, a typical underdeveloped ethnic autonomous region in western China, exhibits a faster aging rate than the national average and distinctive regional features including a high concentration of ethnic minorities, an extensive border, and rugged mountainous terrain. Historically, its per capita medical expenditure has lagged behind the national average, and the foundation of primary healthcare is fragile [3]. The dual challenge of poverty preceding old age and encountering resource limitations faced by Guangxi holds significant representativeness and prevalence in the underdeveloped regions of western China. Thus, commencing from rural communities in Guangxi to investigate the healthcare requirements of the elderly in underprivileged areas bears crucial exemplar significance in addressing elderly care issues in analogous regions.Amid China’s accelerating population aging and rural depopulation, health issues among elderly residents in rural communities of underdeveloped regions have become increasingly prominent, placing heavier economic and caregiving burdens on families [4, 5].

The World Health Organization notes that healthcare needs among older adults exhibit diverse, multilevel characteristics [6]. Research indicates that approximately 264 million older adults in China suffer from at least one chronic disease, accounting for 75.8% of the elderly population [7]. With advancing age, functional decline and the risk of multiple diseases increase. Among those aged 85 and above, the prevalence of multiple diseases reaches 82% [8]. Furthermore, frailty poses a significant global challenge to the health of the elderly. A multinational study indicates a global weighted prevalence of frailty of 10.7% among individuals aged 65 and older [9]. In China, frailty prevalence reaches 10% among community-dwelling seniors aged 60 and older, 25% among those aged 85 and older, and 30% among hospitalized elderly [10]. These health issues are enduring, persistent, and intricate. The piecemeal medical services offered by hospitals are insufficient to adequately tackle them. There is a pressing requirement for a comprehensive health intervention across the entire spectrum. Communities, as the primary milieu for the elderly and the cornerstone of medical and health services, play a vital role in implementing strategies for managing chronic diseases, preventing frailty, and promoting health [11]. Therefore, this study focuses on the community level and defines community-based elderly health management as the evaluation of factors influencing the health of older individuals in the community. Its objective is to steer and support the elderly in embracing effective preventive and intervention measures to realise comprehensive health management initiatives for disease prevention, as well as the preservation and enhancement of elderly health .Research indicates that health management needs assessments enhance resource allocation efficiency, facilitate targeted community health interventions, and improve community healthy aging levels [12].

However, health management needs among older adults exhibit multilevel complexity [13]. Current international research on health management needs for older adults primarily focuses on long-term care requirements [14], healthcare service demands [15], health information management needs [16], or disease-specific health management requirements [17, 18]. Regarding research on the health management needs of older adults and their influencing factors, China has made preliminary progress in this field [19, 20]. Previous studies indicate that factors influencing older adults’ health management needs include age, gender, education level, income level, and chronic disease status [21, 22]. Relevant studies indicate that residents in China’s rural communities generally exhibit lower economic and cultural levels, constrained by traditional beliefs and relatively inadequate health literacy, and insufficient emphasis on health management. They also face challenges such as relatively scarce health management data and an imperfect medical and elderly care service system [23, 24]. However, existing Chinese research primarily focuses on urban or rural communities in developed regions, exhibiting a degree of homogeneity [25]. The majority of current research relies on the conventional Logistic regression technique, which presupposes a linear association between variables and their mutual independence. This assumption hinders the ability to detect potential nonlinear connections and higher-order interrelations within the intricate social context of less developed areas. Furthermore, the traditional approach prioritises statistical inference over the capacity to assess the significance of variables and measure their impacts, thus impeding the provision of a scientific rationale for targeted and prioritised interventions in resource-constrained regions [26].

In response to the aforementioned methodological limitations, machine learning (ML) has been extensively applied in public health, playing a significant role in early disease detection and healthcare demand forecasting [27, 28]. The primary aim is to achieve accurate predictions for sample classification or regression tasks by revealing the underlying patterns within the data, while optimising feature representations and model parameters. Notably, the XGBoost algorithm is extensively employed due to its robust classification performance. The introduction of the SHAP (Shapley Additive Explanations) method from game theory effectively addresses the black box issue associated with machine learning models. This approach facilitates both global and local quantification of the direction and magnitude of each feature’s contribution to the prediction outcomes, thereby providing interpretable decision support that aligns with the actual requirements of underdeveloped regions facing resource constraints in accurately identifying high-risk populations [29].

This study focuses on rural communities in underdeveloped regions of Guangxi, China, to develop a precise predictive model for elderly health management needs using machine learning. The aim is to address the disparity between limited grassroots service capabilities and diverse needs. Key predictive variables are selected through LASSO regression and binary logistic regression models. Nomogram charts are created for quick clinical assessment. Additionally, random forest feature importance analysis and the XGBoost model, along with the SHAP interpretation framework, are employed to delve into the nonlinear driving mechanisms and threshold effects of each influencing factor on health management needs. The study uncovers the intricate determinants of health management needs in less developed areas, offering a scientific foundation for local governments to implement tailored health interventions under resource constraints. This approach supports the effective establishment of a comprehensive life cycle health management service system in underdeveloped ethnic regions and facilitates the realization of the active aging strategy [30].

## Methods

### Study design and participants

The 14 prefecture-level cities in Guangxi were divided into four tiers based on their 2023 per capita GDP. One city was randomly selected from each tier, resulting in four cities. From August 2023 to January 2024, a questionnaire survey was administered to elderly individuals in rural communities across four cities: Beihai, Nanning, Guilin, and Guigang. The survey employed a multi-stage stratified sampling method.Inclusion Criteria: ①Age ≥ 60 years; ②Seniors choosing home-based care; ③Clear consciousness; ④Ability to understand questionnaire questions and respond smoothly with interviewer assistance; and informed consent to participate. ⑤Residents of rural communities in Guangxi who have resided there for a minimum of six months include individuals with rural household registration as well as those with urban household registration who have been long-term residents of these rural areas.Exclusion criteria: ①Mental illness; ②Intellectual or cognitive impairment; ③Significant hearing impairment or language communication barriers. A total of 651 elderly individuals were surveyed, yielding 641 valid responses, for an effective response rate of 98.46%. The dataset was randomly divided into a training set (449 cases) and a validation set (192 cases) at a 7:3 ratio.

### Definition of health management needs

Health management needs refer to the interventions and resources individuals or groups seek to maintain and promote their health. To measure the prevalence of health management needs among the elderly, this study developed a questionnaire based on the Zuluaga-Raysmith (Z-R) model. This questionnaire was created through literature review, theoretical analysis, and expert consultation [31]. Participants were assessed by responding to the statement, “Do you have relevant needs for health management services?” Answering “Yes” counts as 1 point, while answering “No” counts as 0 points. Respondents scoring 1 point are considered to have health management needs.This method has demonstrated strong face validity and discriminant validity across multiple studies concerning the health needs of the elderly [21, 32]. The reliability and validity of the questionnaire are commendable. The overall Cronbach’s α coefficient for the scale is 0.77 (95% CI: 0.75–0.80), with a KMO value of 0.80 and a Bartlett’s test of sphericity yielding χ² = 2413.001 (df = 120, *P* < 0.001). Employing principal component analysis and an orthogonal rotation method, five factors with eigenvalues exceeding 1 were identified, resulting in a cumulative variance explanation rate of 38.5%. The load matrix analysis revealed a model fit index with TLI = 0.901 and RMSEA = 0.054 (90% CI: 0.044–0.065), indicating a robust fit for the factor structure.

### Measurement of other variables

In this study, demographic variables included household registration type (rural, urban), age (60–64 years, 65–69 years,70–74 years, 75 years and above), spouse(yes, no), education level (primary school or below, junior high/vocational school, high school, college/undergraduate, postgraduate or above), occupation (government/public institution staff, enterprise employee, farmer, self-employed, freelancer, unemployed), monthly per capita income (< 800, 800–1499, 1,500–2,999, 3,000 and above), medical insurance type (urban employee medical insurance, urban and rural residents medical insurance, commercial insurance, none), duration of chronic disease (0 years, 1–2 years, 3–4 years, 5–9 years, 10–14 years, 15 years and above), history of critical illness (yes, no). Self-care ability (able to care for oneself, requires moderate assistance from others, unable to care for oneself). Health behavior variables include: Frequency of physical examinations (every six months, once a year, irregularly, rarely), Smoking status (no, quit, yes), Alcohol consumption (no, quit, yes), Insomnia (yes, no), Participation in health activities (frequently, occasionally, rarely, never)Other relevant variables include: relationship with children (excellent, good, average, not very good, poor); social functioning (excellent, good, average, not very good, poor). Individual factors in health management include understanding chronic disease risk factors, actively learning about health management, using mobile phones and other smart devices, measuring health indicators at home, recognising the necessity of regular health checkups, maintaining a positive mindset, and feeling satisfied with one’s current circumstances. Multiple-choice items include: current status of community health management, Channels for acquiring knowledge about diseases, and suggestions for community health management.

### Statistical analysis

This study utilised SPSS 26.0 and R 4.5.1 for statistical analysis of the research data. Categorical variables were presented as frequency (n) and percentage (%), with the chi-square (χ²) test employed for intergroup comparisons. Variances in individual factors were assessed through the independent sample t-test. A combined screening strategy involving Logistics Regression analysis and LASSO regression was employed. Binary Logistic regression analysis was performed on the training set to identify potential predictive factors with *P* < 0.05. Concurrently, LASSO regression (L1 regularization) was utilised to address multicollinearity and further refine the variable space. In the LASSO model, the bias -λ curve was generated through 5-fold cross-validation. To enhance model sensitivity and prevent over-penalisation that could lead to the exclusion of significant features, the λ value corresponding to the minimum cross-validation error (λ.min) was selected. Ultimately, the union of the screening results from single-factor Logistic regression and LASSO regression constituted the core predictor set. Using this factor set, a binary Logistic regression model was developed, and a visualised column chart was created. The core predictive factors identified were integrated into the random forest (RF) and extreme gradient boosting (XGBoost) models. The model was retrained employing a grid search combined with a cross-validation strategy, adjusting the node size (nodesize), maximum number of nodes (maxnodes), and the number of trees (ntree) to optimise the performance of the random forest (RF). By modifying the maximum tree depth (max_depth), subsample ratio (subsample), column subsampling ratio (colsample_bytree), and decreasing the number of iterations while restricting the tree depth, the XGBoost model can be optimised to attain peak performance.

The dataset was stratified and randomly divided into training and independent validation sets for internal validation. Within the training set, alongside the aforementioned hyperparameter tuning, the 95% confidence interval of the Brier score was computed using 1000 bootstrap resampling. The generalisation capability of the logistic regression, random forest, and XGBoost models was assessed using a completely independent validation set. This study specifically employed the SHAP (SHapley Additive exPlanations) framework to conduct an in-depth analysis of the XGB model. By calculating the SHAP values for each feature, bar charts illustrating feature importance, summary plots, and dependence plots were generated to visually represent the direction, intensity, and non-linear relationships of each predictive factor concerning health management needs. Concurrently, the feature importance ranking of the random forest was compared to verify the robustness of the key drivers. Model performance was evaluated using multiple indicators: the area under the receiver operating characteristic (ROC) curve (AUC) assessed discrimination ability. Prediction consistency was evaluated through calibration curves and Brier scores; clinical net benefit was assessed using decision curve analysis (DCA), and accuracy, sensitivity, specificity, precision and F1 score were calculated from the confusion matrix to provide a comprehensive evaluation of the model’s overall performance.

## Results

### Participant characteristics

This study included 641 elderly individuals, among whom 281 (43.84%) had health management needs. Statistically significant differences in health management needs were observed among elderly individuals with varying household registration status, age, education level, occupation, per capita monthly income, frequency of physical examinations, health activities, relationship with children, and social functioning (*P* < 0.05)(Table B1**)**. The study divided the data into a training set (449 cases) and a validation set (192 cases). No statistically significant differences were observed between the training and validation groups in variables related to health management needs (household registration status, age, marital status, educational attainment, et al.) (*P* > 0.05). Statistically significant differences were only observed for smoking status and alcohol consumption (*P* < 0.05) (Table 1).

**Table 1:** Comparison of baseline characteristics between the training group and the validation group among individuals aged 60 years and over in Guangxi (*N* = 641)

Variables	Training dataset (*n* = 449)	Validation dataset (*n* = 192)	X^2^	*P*
Household Registration Status			2.907	0.088
Rural	327 (72.8)	127 (66.1)		
Urban	122 (27.2)	65 (33.9)		
Age (years)			6.305	0.098
60～	108 (24.1)	43 (22.4)		
65～	129 (28.7)	74 (38.5)		
70～	115 (25.6)	42 (21.9)		
≥75	97 (21.6)	33 (17.2)		
Spouse			1.220	0.269
No	129 (28.7)	47 (24.5)		
Yes	320 (71.3)	145 (75.5)		
Educational level			2.708	0.608
Elementary school and below	233 (51.9)	97 (50.5)		
Junior High/Vocational School	104 (23.2)	40 (20.8)		
High School	52 (11.6)	31 (16.1)		
College/Undergraduate	43 (9.6)	18 (9.4)		
Bachelor’s degree or above	17 (3.8)	6 (3.1)		
Occupation			3.934	0.559
Party and government organs/public institutions personnel	60 (13.4)	26 (13.5)		
Enterprise employees	41 (9.1)	20 (10.4)		
Farmers	230 (51.2)	90 (46.9)		
Self-employed	42 (9.4)	21 (10.9)		
Freelancer	33 (7.3)	21 (10.9)		
Unemployed	43 (9.6)	14 (7.3)		
Monthly Income per Capita (CNY)			2.684	0.443
<800	77 (17.1)	41 (21.4)		
800～	137 (30.5)	49(25.5)		
1500～	165 (36.7)	69 (35.9)		
≥3000	70 (15.6)	33 (17.2)		
Health Insurance Type			2.515	0.473
Urban Employee Medical Insurance	46 (10.2)	25 (13.0)		
Urban and Rural Residents’ Medical Insurance	375 (83.5)	155 (80.7)		
Commercial Insurance	10 (2.2)	2 (1.0)		
None	18(4.0)	10(5.2)		
Health Management Needs			0.001	0.979
No	231 (51.4)	99 (51.6)		
Yes	218 (48.6)	93 (48.4)		
Duration of chronic disease (years)			2.894	0.716
None	155 (34.5)	63 (32.8)		
0～	67 (14.9)	24 (12.5)		
3～	88 (19.6)	43 (22.4)		
5～	105(23.4)	42(21.9)		
10～	18(4.0)	12(6.3)		
≥15	16 (3.6)	8 (4.2)		
History of critical illness			0.001	0.972
No	384 (85.5)	164 (85.4)		
Yes	65 (14.5)	28 (14.6)		
Health Check Frequency			2.481	0.479
Every six months	46 (10.2)	24 (12.5)		
Once a year	136 (30.3)	63 (32.8)		
Irregular	214 (47.7)	79 (41.1)		
Rarely	53 (11.8)	26 (13.5)		
Smoking			10.420	<0.01
No	243 (54.1)	127 (66.1)		
Quit	107 (23.8)	26 (13.5)		
Yes	99 (22.0)	39 (20.3)		
Alcohol consumption			8.677	<0.05
No	224 (49.9)	118 (61.5)		
Quit	98 (21.8)	26 (13.5)		
Yes	127 (28.3)	48 (25.0)		
Insomnia Status			0.391	0.532
No	283 (63.0)	116 (60.4)		
Yes	166 (37.0)	76 (39.6)		
Healthy Activities			1.078	0.782
Frequently	53 (11.8)	19 (9.9)		
Occasionally	120 (26.7)	58 (30.2)		
Rarely	127 (28.3)	53 (27.6)		
Never	149 (33.2)	62 (32.3)		
Self-care ability			0.271	0.873
Able to care for oneself	364 (81.1)	156 (81.3)		
Requires appropriate assistance from others	76 (16.9)	31(16.1)		
Unable to care for oneself	9 (2.0)	5 (2.6)		
Children’s Affection			6.699	0.153
Very Good	97 (21.6)	53 (27.6)		
Good	235 (52.3)	93 (48.4)		
Average	96 (21.4)	31 (16.1)		
Not very good	13 (2.9)	9 (4.7)		
Poor	8 (1.8)	6(3.1)		
Social Functioning			6.474	0.166
Very Good	72 (16.0)	43(22.4)		
Good	200(44.5)	90(46.9)		
Average	140 (31.2)	45 (23.4)		
Not very good	26 (5.8)	11 (5.7)		
Poor	11 (21.4)	3(1.6)		

### Variable selection

**Fig. 1:**
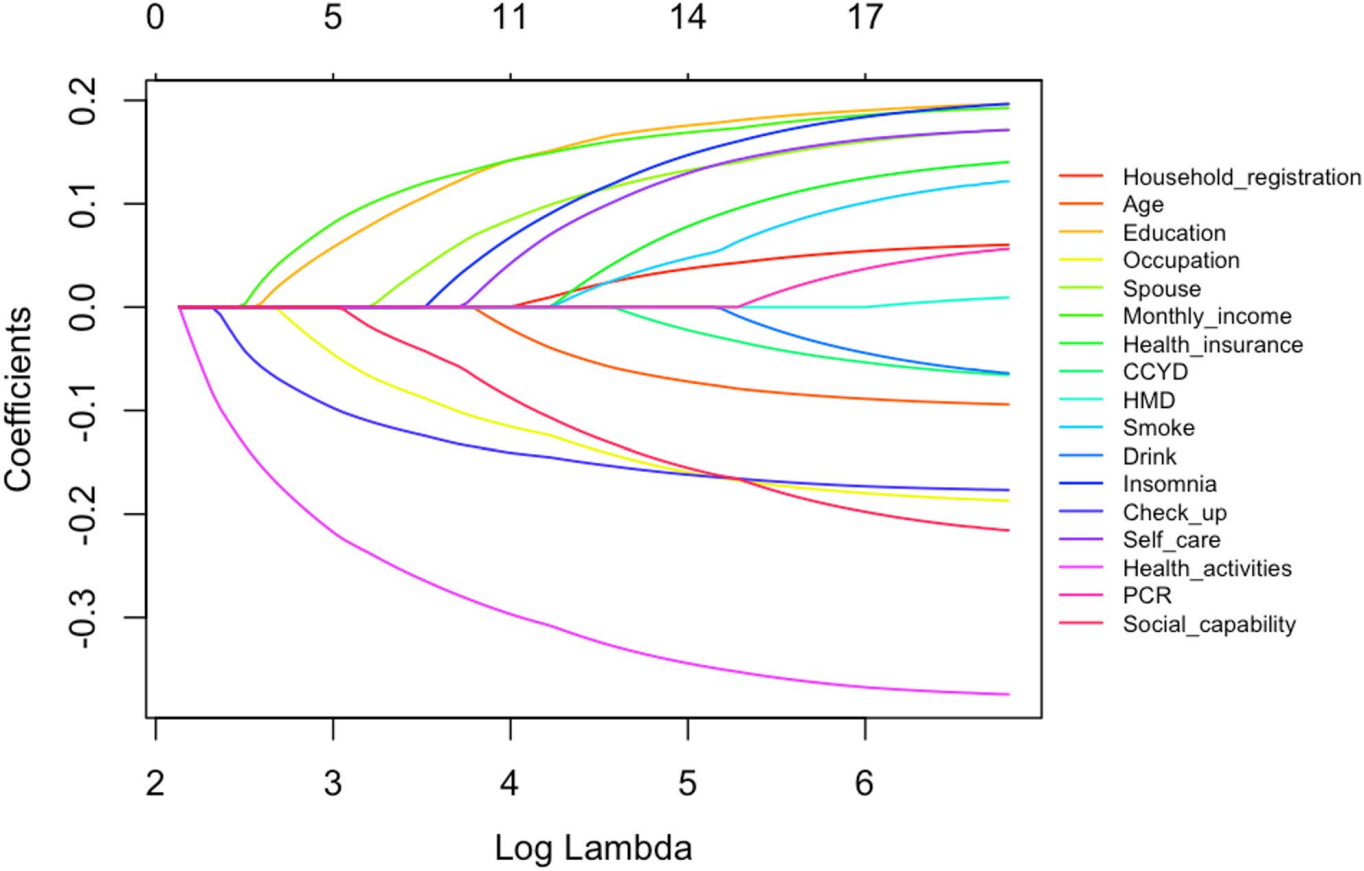
The study utilised the LASSO regression model to identify significant influencing factors. Figure illustrates the regression coefficient curve produced from the log(λ) sequence. With the rise in regularization strength (λ), the coefficients of individual variables diminish towards 0. The non-zero coefficients were obtained using the optimal λ value (lambda.min) to ascertain the variables for inclusion in the model

**Fig. 2:**
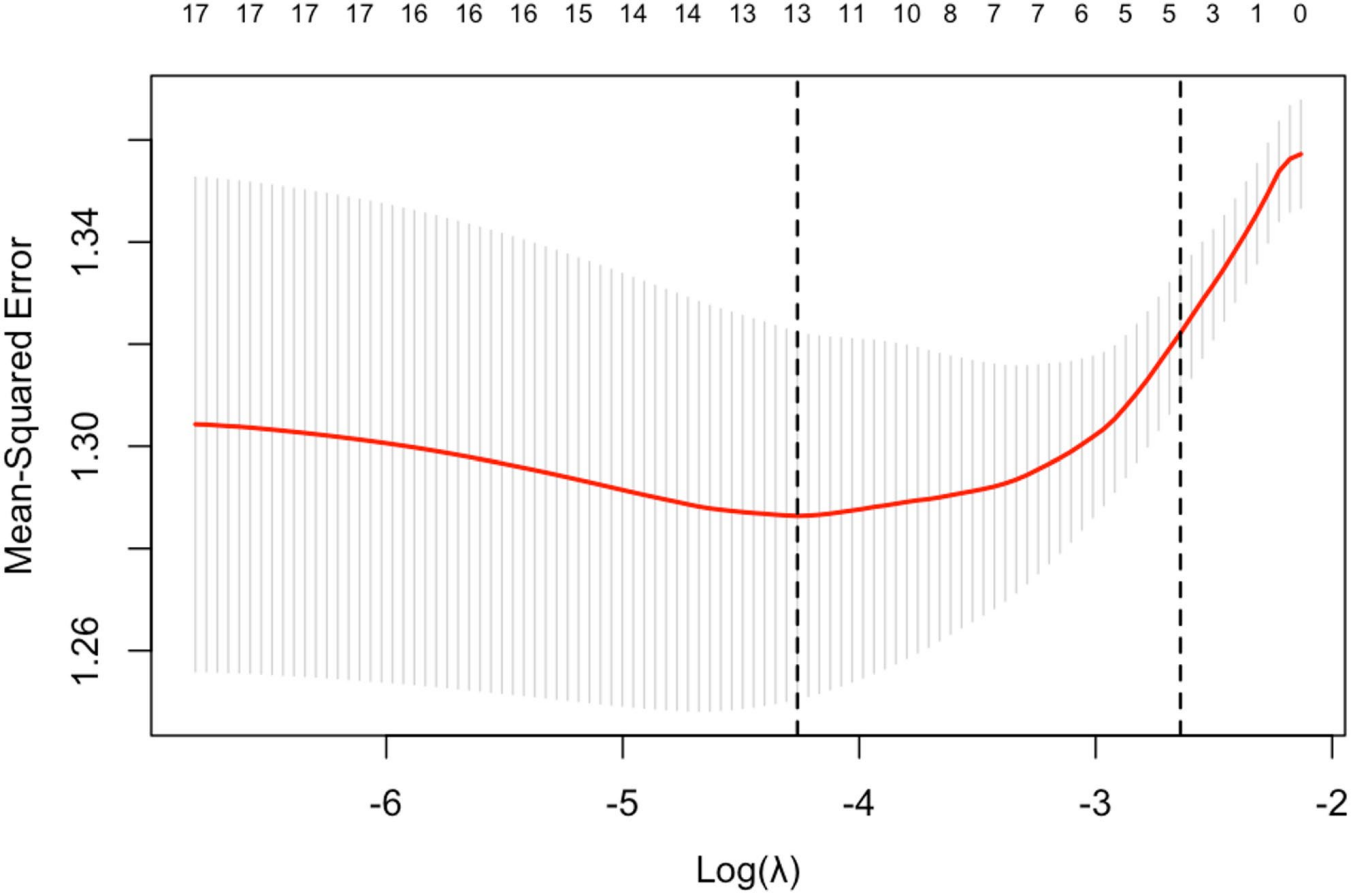
The study displays the outcomes of 5-fold cross-validation, featuring a binomial deviance curve to ascertain the optimal λ value within the LASSO model. The dashed lines depicted in the graph represent lambda.min (the lambda value minimising deviation) and lambda.1se (the lambda value based on a more conservative 1 standard error criterion)

This study utilised LASSO regression in conjunction with logistic regression models to identify relevant variables. An iterative analysis was performed employing the 5-fold cross-validation method, which adjusted for sample size adaptability. The LASSO regression coefficient path diagram (Fig. 1) illustrates two key regularisation parameters, λ values (lambda.min and lambda.1se); each curve depicts the trajectory of coefficient changes as λ increases for various variables. The significance of a variable is indicated by the timing of its coefficient’s reduction to zero, with more important variables exhibiting a later decline. The LASSO cross-validation error graph (Fig. 2) reveals that as λ increases, the model’s regularisation intensity enhances, leading to a gradual shrinkage of regression coefficients and a decrease in the number of included predictor variables. Based on lambda.min, which minimises cross-validation bias, 13 variables were ultimately selected: Household registration, Age, Education, Occupation, Spouse, Monthly income, Health insurance, Smoke, Insomnia, Check up, Self care, Health activities, and Social capability. Through binary logistic regression (*P* < 0.05), 8 variables were identified: Check up, Education, Health activities, Household registration, Monthly income, Occupation, Parent-child relationship, and Social capability. The union of these variables included: Check up, Health activities, Monthly income, Education, Occupation, Social capability, Household registration, Parent-child relationship, Age, Spouse, Health insurance, Smoke, Insomnia, and Self care, which were incorporated into the subsequent modelling analysis.

### Construction of logistic regression risk prediction model

**Fig. 3:**
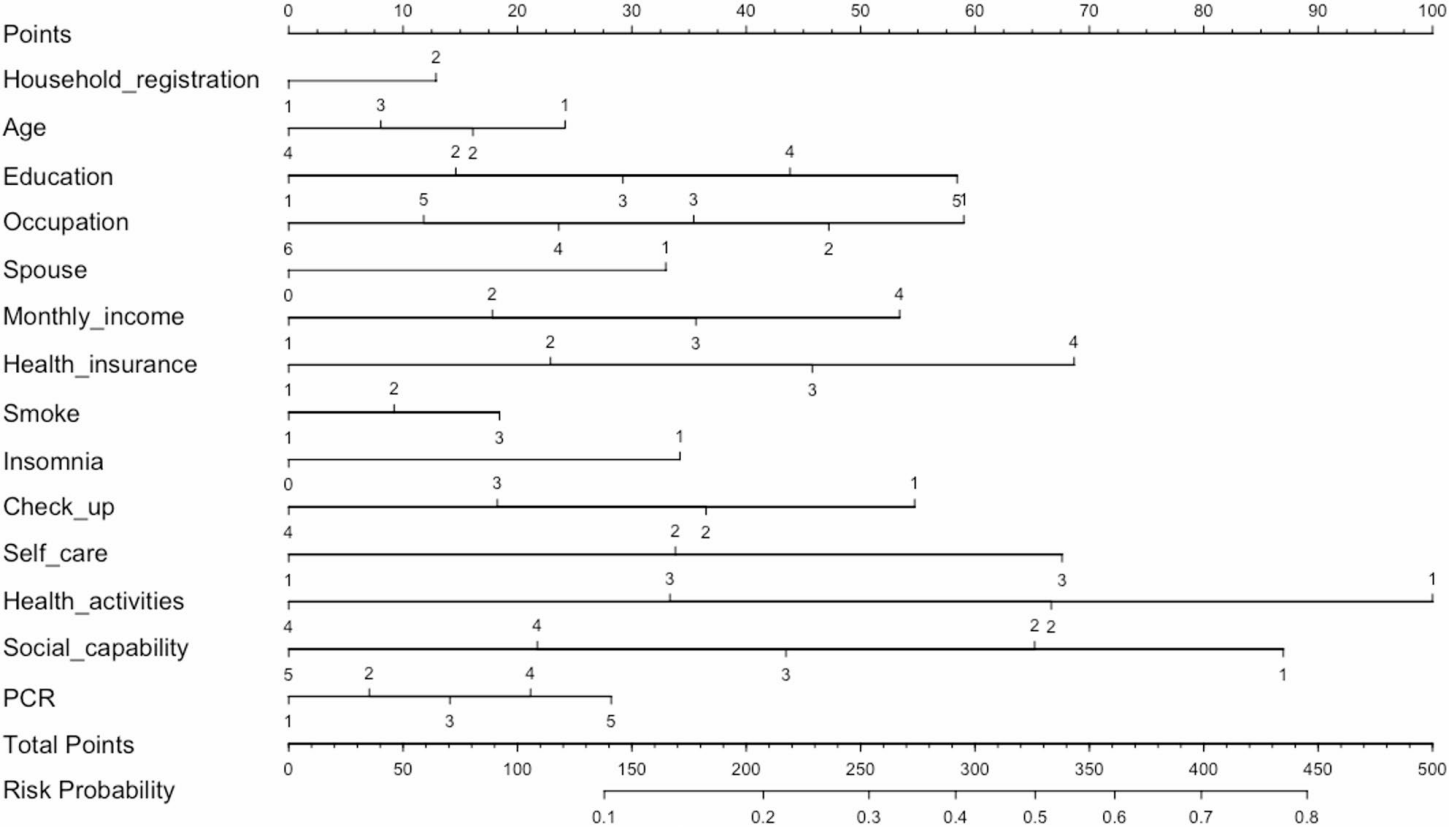
Nomogram of predictors for elderly health management needs

This study incorporated 14 independent factors to develop a predictive model. The findings from the multivariate logistic regression analysis revealed that monthly income, health activities and social capability significantly influenced the health management needs of the elderly (Table B2). Variable assignment is detailed in (Table B3). The model was constructed employing the “rms” package in RStudio, while the nomogram illustrating the health management needs of the elderly was generated using the “nomogram” package (Fig. 3).

### Building the random forest risk prediction model

**Fig. 4:**
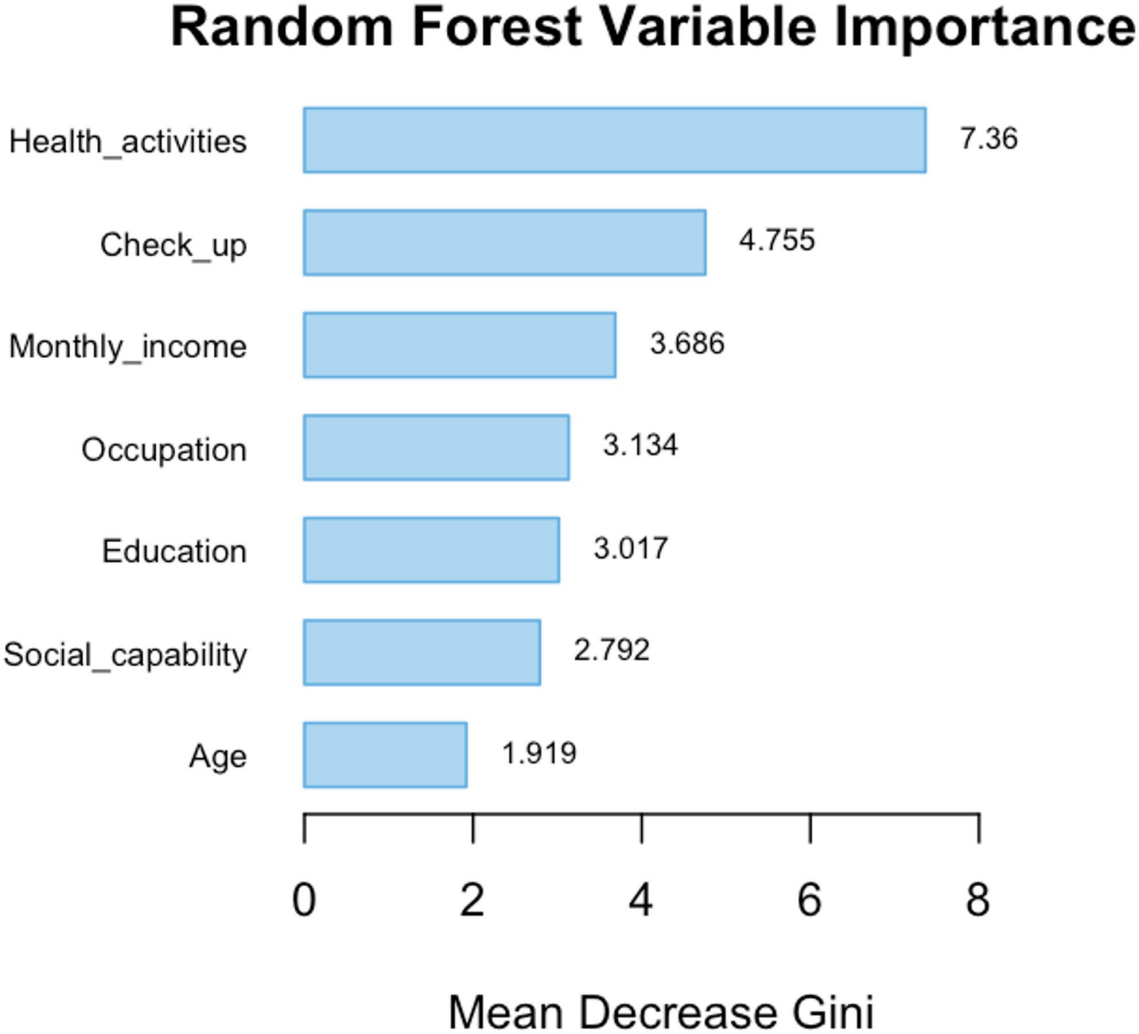
Ranking of key factors influencing health management needs among the elderly in the random forest model

A random forest model was developed using the training set data. The model exhibited optimal performance when mtry was set to 3 and ntree to 500. The analysis of out-of-bag (OOB) error convergence indicates that the error stabilised following the construction of the 211th tree (Fig A1). Based on the mean Gini coefficient, the importance of the seven variables was ranked (Fig. 4). The ranking of the factors influencing health management needs is as follows: health activities, regular physical examinations, per capita monthly income, occupation, education level, social capability, and age.

### Building the XGBoost risk prediction model

**Fig. 5:**
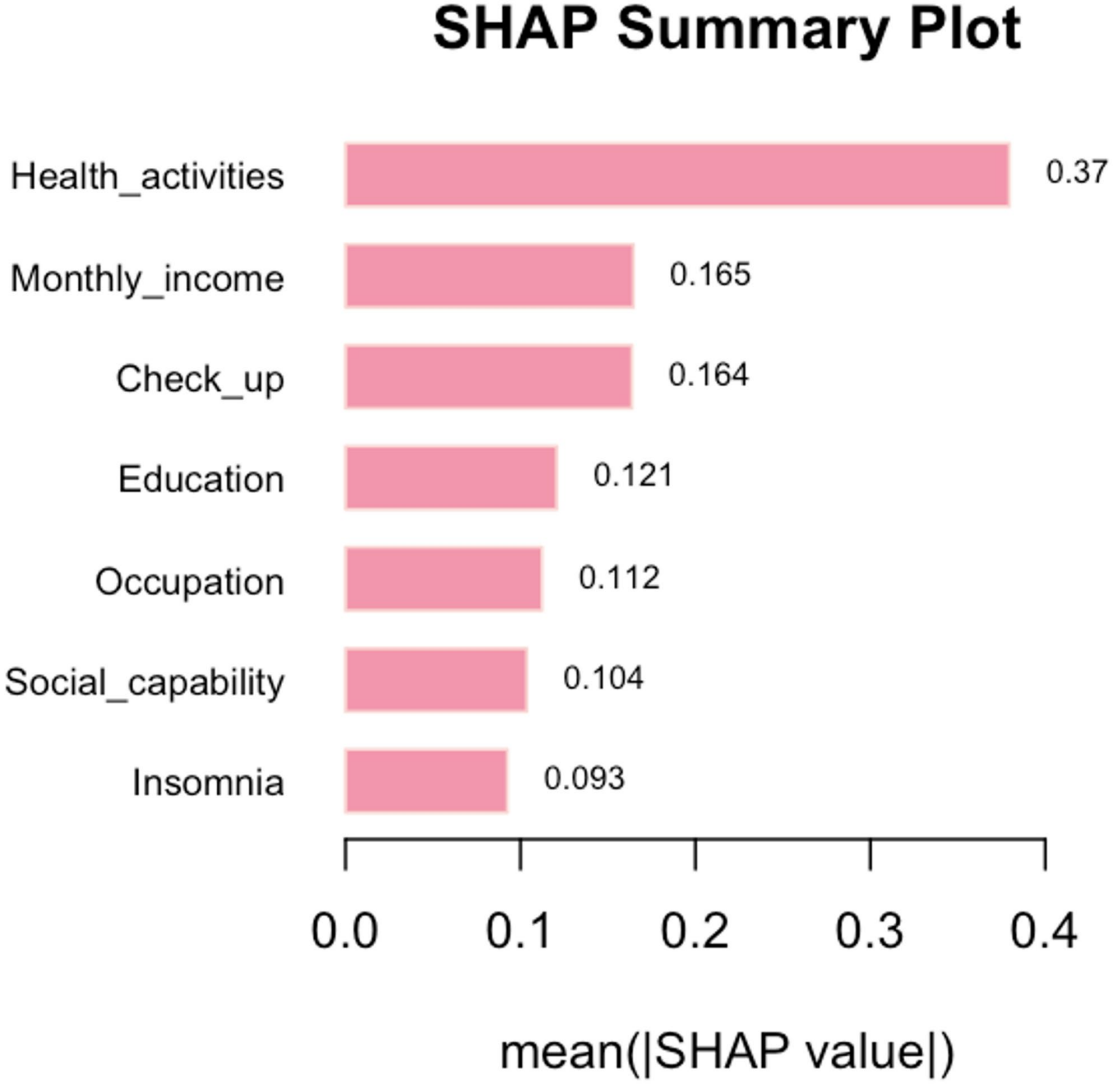
Feature importance of the XGBoost model influencing elderly health management needs

**Fig. 6:**
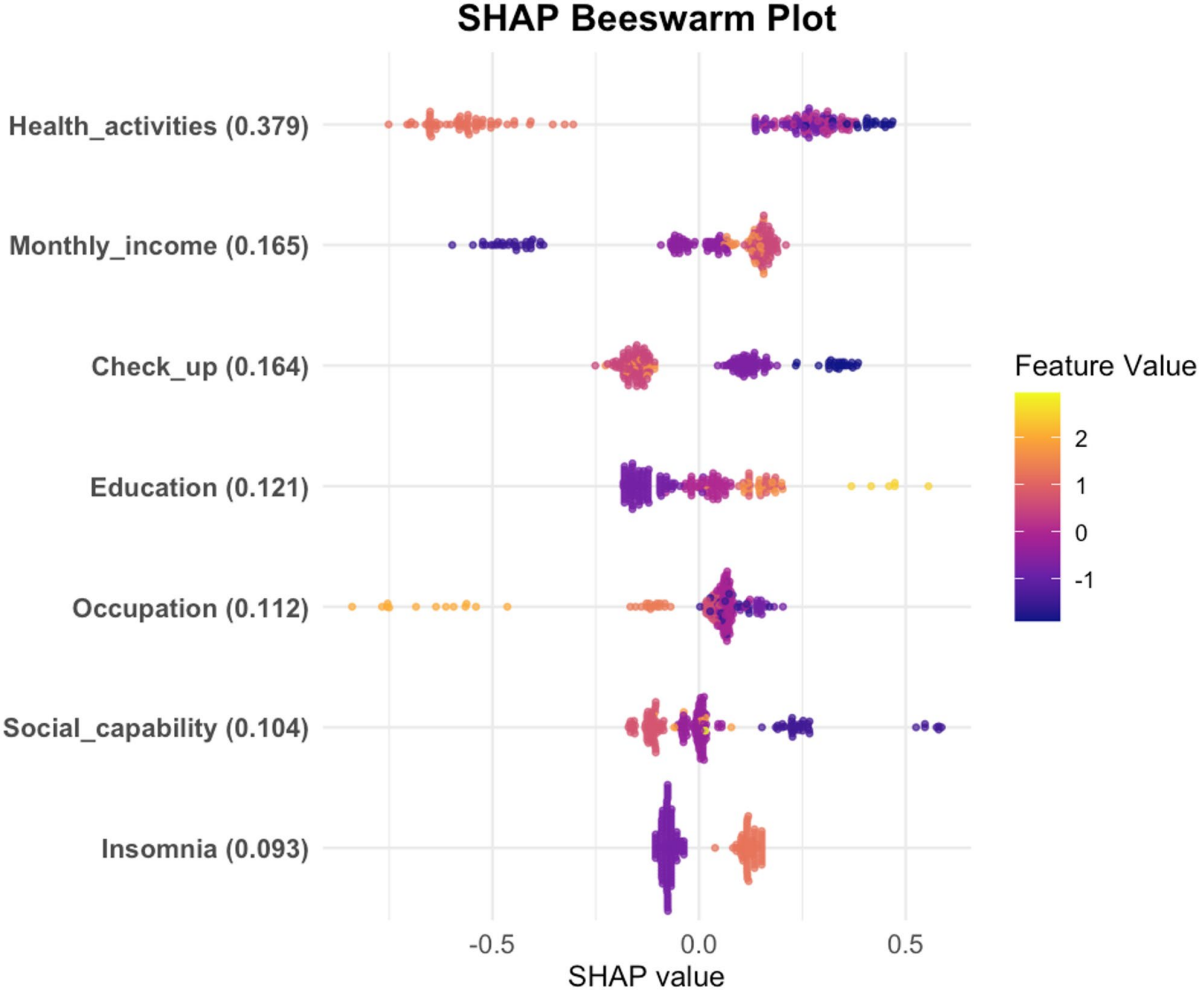
SHAP distributions for key features influencing elderly health management needs in the XGBoost model

**Fig. 7:**
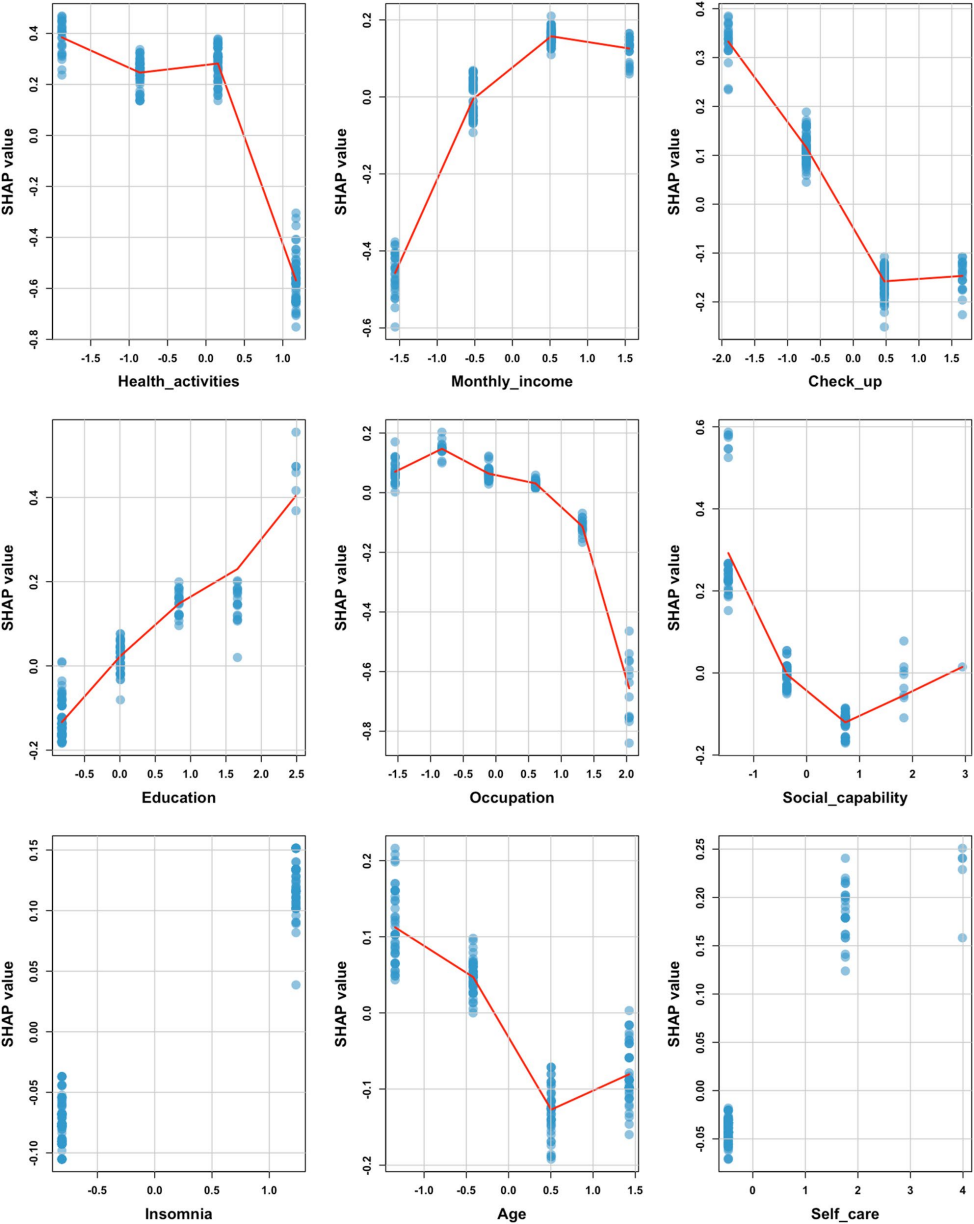
SHAP value relationships among key factors influencing elderly health management needs in the XGBoost model

This research utilises the XGBoost model to identify the determinants of health management requirements among the elderly. The initial graph is centred on the mean SHAP. The analysis of feature importance reveals that health-related activities, monthly income per capita, frequency of medical check-ups, educational attainment, and occupational status are the primary factors influencing the health management needs of older individuals (Fig. 5). A joint impact assessment was performed on these five variables, demonstrating that individuals with a high school education, those earning between 1500 and 3000 yuan per month with a high school education, those lacking regular medical check-ups with a high school education, and those earning 800–1500 yuan per month with a high school education exhibit elevated health management requirements (Table 2). The subsequent diagram is constructed using a beeswarm plot. The encoded values for health-related activities and medical check-up frequency variables are notably higher, indicating lower engagement. Notably, elevated encoded values correspond to markedly positive SHAP values, underscoring that insufficient participation in health-related activities and infrequent medical check-ups strongly drive health management needs, whereby reduced frequency correlates with heightened demand. Moreover, increased income, higher educational attainment, enhanced social functioning, and experiencing insomnia all substantially elevate the demand, whereas holding a traditional or fixed job status significantly diminishes it(Fig. 6).The third figure, derived from a dependence plot, illustrates that elevated education and income levels, infrequent engagement in health activities and medical check-ups, insomnia, and strong social functioning positively influence the necessity for health management. Conversely, increased self-care proficiency and engagement in traditional occupations or non-permanent employment substantially diminish this demand (Fig. 7).

**Table 2:** Analysis of the synergistic effects of five key variables on health management needs

Interaction terms	Estimate	Std.Error		Z value	*P* -value
Education3:Occupation2	7.383	2.463	2.997		0.003
Education3:Monthly_income3	5.110	2.073	2.465		0.014
Education3:Check_up4	5.392	2.215	2.434		0.015
Education3:Monthly_income2	4.359	2.039	2.138		0.033

### Performance comparison of three predictive models

**Fig. 8:**
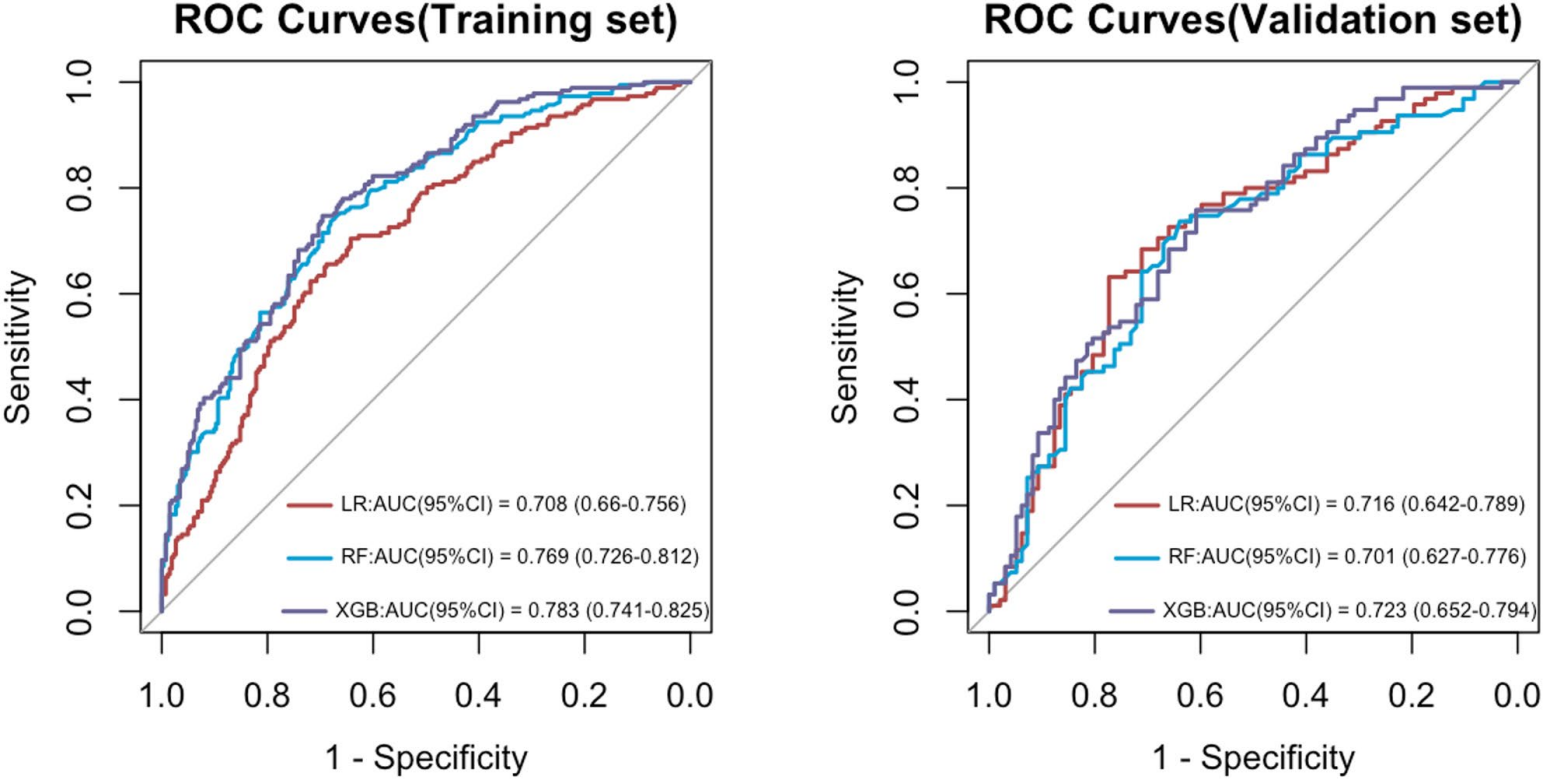
Comparison of AUC between training and validation sets for three models of elderly HMN

**Fig. 9:**
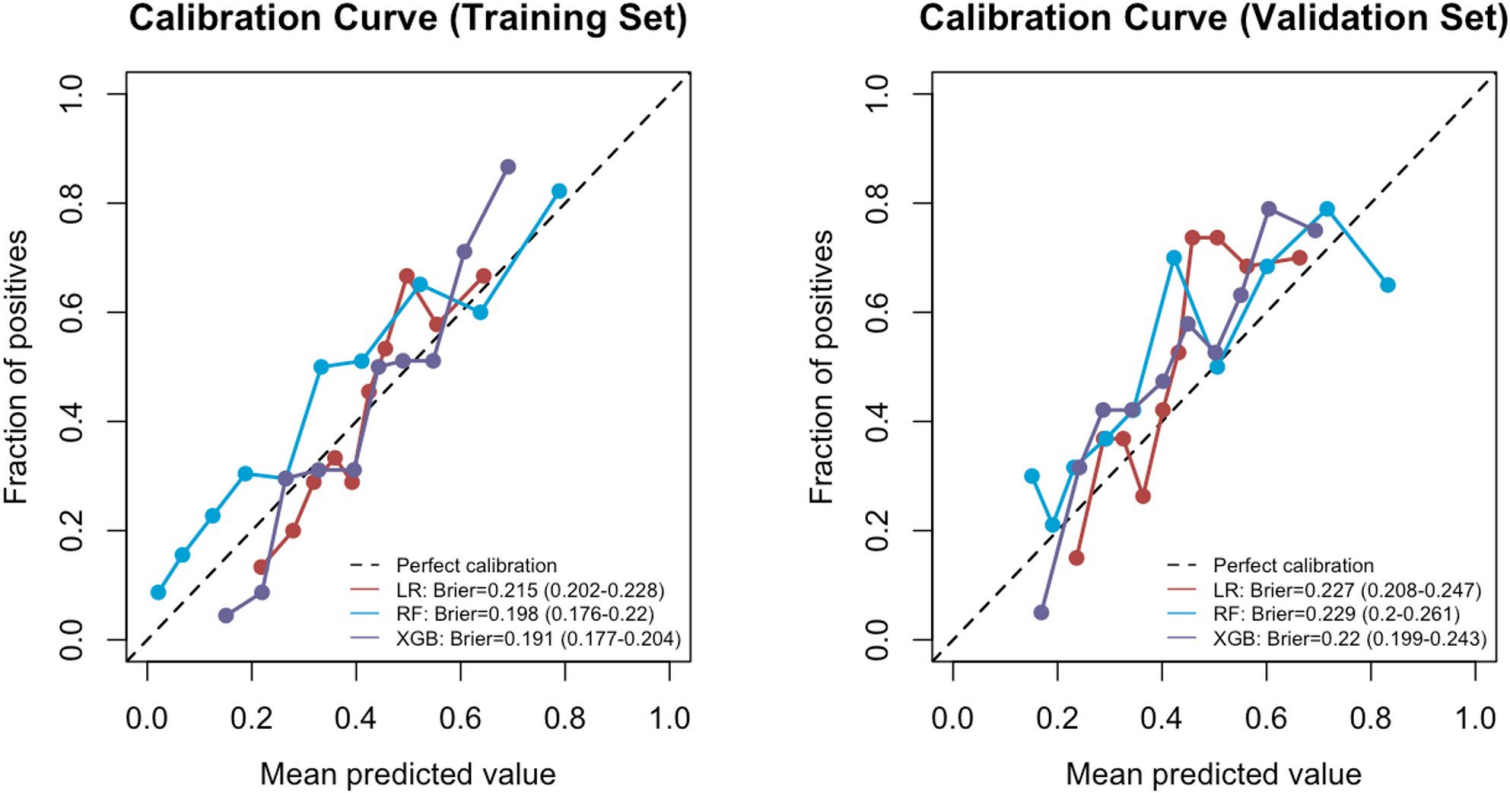
Comparison of calibration curves for three models of elderly HMN across training and validation sets

**Fig. 10:**
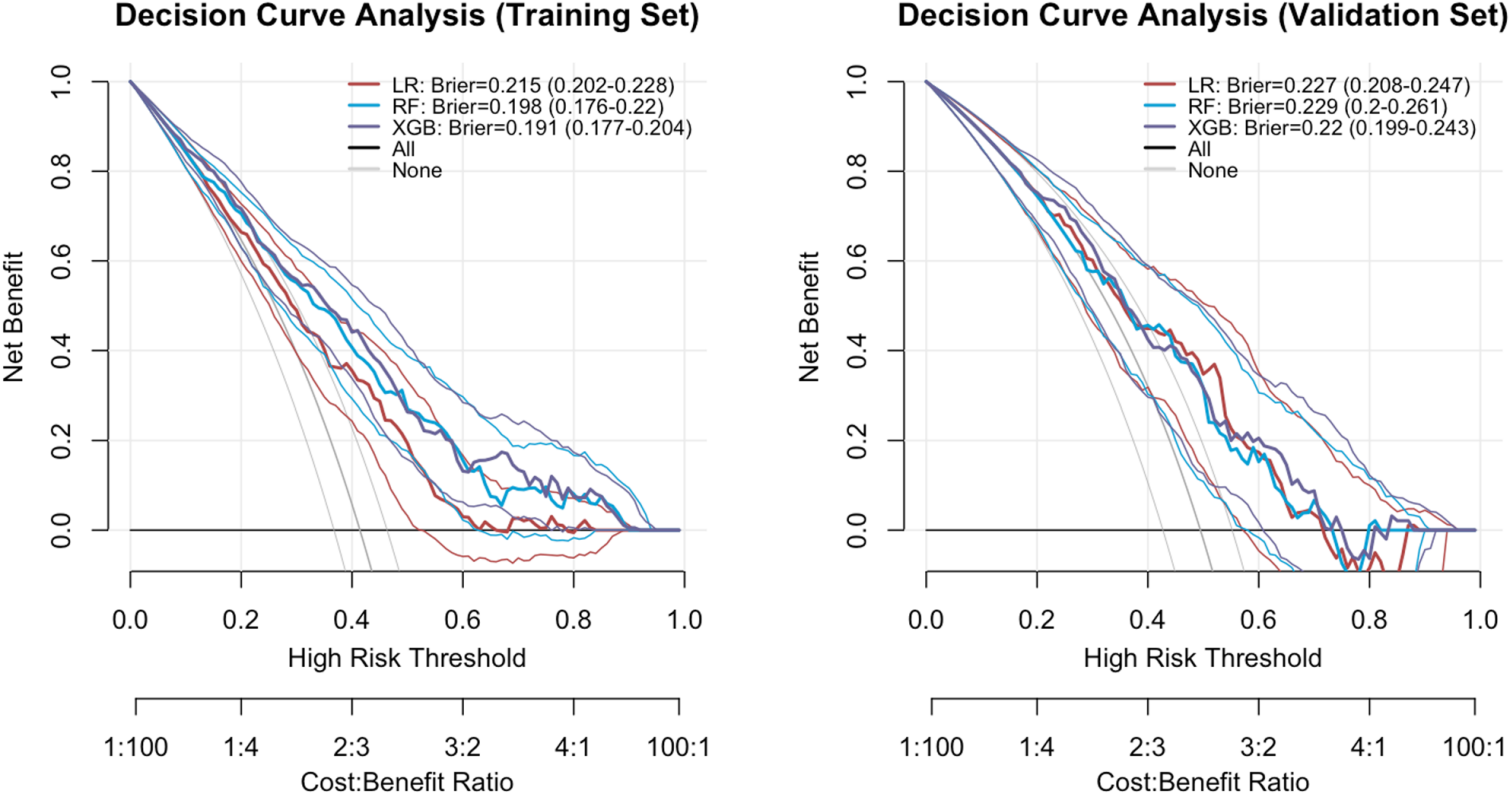
Comparison of DCA curves for three models on training and validation sets for elderly HMN

This study utilised Logistic Regression (LR), Random Forest (RF), and XGBoost (XGB) as three machine learning models to develop a predictive model for the health management needs of the elderly. The area under the curve (AUC) for each model ranged from 0.6 to 1.0. The discriminative efficacy, calibration, and clinical net benefit of each model were thoroughly assessed using ROC curves (Fig. 8), calibration curves (Fig. 9), and decision curve analysis (DCA, Fig. 10).Table 3 presents the accuracy, sensitivity, specificity, precision, F1 value, and Brier score for the three models in both the training and validation sets. Considering the overall performance across these sets, the XGBoost model exhibited the highest predictive efficacy, with its AUROC surpassing that of the other models. It demonstrated exceptional discriminative ability in predicting the health management needs of the elderly.The AUC in the training set was 0.783 (95% CI 0.741–0.825), with Accuracy at 0.695, Sensitivity at 0.511, Specificity at 0.825, Precision at 0.674, F1 value at 0.581, and Brier score at 0.191. In the validation set, the AUC was 0.723 (95% CI 0.652–0.794), Accuracy at 0.656, Sensitivity at 0.505, Specificity at 0.804, Precision at 0.716, F1 value at 0.593, and Brier score at 0.22. The Random Forest model’s performance in the training set (Accuracy = 0.697, Sensitivity = 0.457, Specificity = 0.867) was comparable to XGBoost, but its performance in the validation set (Accuracy = 0.615, Sensitivity = 0.463, Specificity = 0.763) slightly decreased. Logistic Regression exhibited slightly inferior performance compared to the tree models across all metrics.

**Table 3:** Performance metrics of various machine learning models in predicting HMN across training and validation sets

Model	Dataset	Accuracy	Sensitivity	Specificity	Precision	F1	Brier
LR	Training	0.648	0.366	0.848	0.630	0.463	0.215
LR	Validation	0.630	0.389	0.866	0.740	0.510	0.227
RF	Training	0.697	0.457	0.867	0.708	0.556	0.198
RF	Validation	0.615	0.463	0.763	0.657	0.543	0.229
XGB	Training	0.695	0.511	0.825	0.674	0.581	0.191
XGB	Validation	0.656	0.505	0.804	0.716	0.593	0.220

### Comparison of individual health management factor scores

Independent samples t-test results indicate statistically significant differences (*P* < 0.05) in individual health management factor scores among older adults based on varying levels of understanding chronic disease risk factors, proactive learning of health management knowledge, use of smartphones and other smart devices, conducting health indicator measurements at home, maintaining a positive mindset, and satisfaction with current living conditions(Table 4).

**Table 4:** Comparative analysis of individual factor scores for HMN among the elderly Points, x ± s

Category	*N*	Disease Awareness	HMN knowledge	Smart Devices	Measuring instrument	Checkups	Positive mindset	Satisfied with life
HMN	281	3.17 ± 0.92	3.21 ± 1.01	3.01 ± 1.13	3.19 ± 1.04	3.69 ± 0.90	3.84 ± 0.80	3.76 ± 0.80
Non-HMN	360	2.94 ± 0.86	2.86 ± 0.89	2.72 ± 1.04	2.76 ± 0.91	3.57 ± 0.90	3.71 ± 0.78	3.45 ± 0.79
t		3.231	4.706	3.352	5.495	1.645	2.040	4.966
P		< 0.005	< 0.001	< 0.005	< 0.001	0.100	<0.05	< 0.001

### Current status of community health management and elderly recommendations

This study employed multiple-choice items to assess the current state of community health management in rural areas and gather suggestions from the elderly regarding community health initiatives. Among the 315 respondents, 49.14% reported that their communities maintain personalized health records for the elderly(X^2^= 44.133, *P* < 0.001). 30.58% (196 individuals) indicated that their communities regularly provide complimentary health consultation services (X^2^ = 82.177, *P* < 0.001). 121 respondents (18.88%) lived in communities that frequently organized health education campaigns; 379 respondents (59.13%) considered regular physical examinations vital; 267 respondents (41.65%) believed their communities valued or highly valued the mental well-being of the elderly; 190 respondents (29.64%) were somewhat or highly proactive in learning health management knowledge. The three primary channels through which older adults acquire disease knowledge are: television and radio (58.66%), sharing among friends and relatives (51.33%), and disease science outreach conducted by the community (31.05%). The three primary health management measures seniors believe communities should implement: regular health monitoring and screening (80.66%), enhanced health management education (69.42%), and targeted chronic disease management services (68.8%). The three main aspects of the community health management environment that older adults believe need improvement are elderly care facilities (77.54%), convenience facilities (65.52%), and environmental hygiene (57.10%) (Table 5).

**Table 5:** Current status of community health management and elderly recommendations

Variable	Content	*n*(%)
Current Status of Community Health Management	Establishment of Personalized Health Records for the Elderly	315 (49.14)
	Providing Free Health Education Outreach Activities for the Elderly	196 (30.58)
	Community Emphasis on the Spiritual Well-being of the Elderly	267 (41.65)
Channels for acquiring disease knowledge	Television and radio	376 (58.66)
	Sharing with Friends and Family	329 (51.33)
	Community Disease Education	199 (31.05)
Health Management Improvement Directions	Regular Health Monitoring and Screening	517 (80.66)
	Enhance Health Management Education	445 (69.42)
	Provide Targeted Chronic Disease Management Services	441 (68.8)

## Discussion

This study developed a predictive model of health management needs among elderly residents in rural communities of underdeveloped regions in China, incorporating standardized variables including demographic, economic, and sociological factors, as well as health behaviors. The key factor of this study lies in selecting rural communities in the typical underdeveloped area of China - Guangxi, which also has the characteristics of a minority region. Machine learning algorithms are used to determine the influencing factors of health management needs. This provides reference materials for relevant departments to improve targeted health management models.

The findings of this study indicate that the demand for health management among elderly individuals in rural communities stands at 43.84%. Compared with previous research, this figure is lower than the 83.33% reported by Yu Xiaoli et al. [33] among 966 elderly individuals undergoing physical examinations and the 67.32% reported by Dong Fen et al. [34] among elderly residents in urban communities. However, it is higher than the 35.97% reported by Li Jun et al. [35]. The survey of middle-aged and elderly farmers in mountainous rural areas found a 35.97% rate. This indicates relatively weak health management awareness among elderly individuals in Guangxi’s rural communities, suggesting the need to strengthen health management education and outreach targeting rural seniors and to establish and improve rural community health management service systems.

This study employed Lasso regression combined with Logistic regression to identify key variables. It systematically compared three machine learning models—Logistic Regression (LR), Random Forest (RF), and XGBoost—in predicting elderly health management needs, while evaluating their decision-making capabilities. Results indicated that the XGB model demonstrated superior predictive performance, with high accuracy, stability, and reliability [36, 37]. The model exhibited an AUC of 0.783 (95% CI: 0.741 - 0.825) in the training set and 0.723 (95% CI: 0.652 - 0.794) in the validation set. Additionally, it demonstrated outstanding performance across various metrics such as Accuracy, Sensitivity, Specificity, Precision, F1 score, and Brier value. According to a study, an AUC value exceeding 0.7 suggests excellent detection capabilities [38].

The analysis using SHAP revealed that the most influential factors influencing the health management requirements of elderly individuals included engagement in health activities, average monthly income, regularity of physical examinations, level of education, and occupation. Furthermore, the interaction analysis highlighted that elderly individuals with a high school education who were employed in enterprises, those with a similar educational background and a monthly income ranging from 1500 to 3000 yuan, individuals with a high school education lacking regular physical check-ups, and those with a high school education and an income between 800 and 1500 yuan exhibited higher health management needs. This study employed a combination of Lasso regression and logistic regression to forecast and sift through 14 variables. The SHAP explainability analysis revealed that inadequate engagement in health activities, stronger social functioning, poor self-care ability, insomnia, increased monthly income, elevated educational attainment, employment in government agencies or public institutions, employment in enterprises, and infrequent physical examinations were associated with an increased likelihood of requiring health management.

Despite Guangxi being a region with a significant population of ethnic minorities, the results from the single-factor analysis and LASSO screening in this study indicated that the “ethnicity” variable did not feature in the final model (*P* > 0.05). This may be attributed to the current rural revitalisation and health poverty alleviation policies in our country, which have led to a high degree of equality in medical security systems and basic public health service standards across different ethnic groups. Consequently, the direct effects associated with ethnic identity have been mitigated by socioeconomic status (SES) factors such as “income,” “education,” and “occupation.”

Subtle distinctions exist in the specific hierarchy of influential factors between the Random Forest (RF) and XGBoost models. The RF model assigns the highest importance to the “physical examination” variable, whereas the XGBoost model accentuates the significance of “monthly income.” This discrepancy arises not from conflicting outcomes but from the distinct evaluation mechanisms and data acquisition traits of the two algorithms. Despite minor fluctuations in ranking, the fundamental sets of factors pinpointed by both models exhibit substantial overlap, affirming the robustness of these pivotal determinants. The healthcare requirements of elderly residents in rural underserved regions are shaped by structural limitations and individual variances.

Research has indicated that older individuals lacking adequate physical activity exhibit reduced health management requirements, contradicting the findings of Li Yichang et al.‘s study [39]. This discrepancy may stem from the heightened susceptibility of inactive elderly individuals to physical function deterioration, as their existing lifestyle fails to fulfil health maintenance needs, prompting a greater inclination towards seeking systematic health management services. Consequently, the likelihood of transitioning health management needs is notably elevated. A Thai study suggests that a robust community network can foster proactive health behaviours among the elderly [40].Elderly individuals with robust social functions typically possess a more comprehensive social support network and enhanced avenues for social interaction. This facilitates their access to health-related information and resources through interpersonal communication, thereby lowering the cognitive threshold and acquisition costs associated with health management services.

Furthermore, active social participation heightens their awareness of personal health status, while group norms and peer influence further bolster their commitment to health management. This phenomenon promotes the transformation of needs from latent to explicit, aligning with the findings of Guan Yan Tong et al. [21]. Insomnia, a prevalent sleep disorder among the elderly, adversely impacts their daytime functioning and long-term health, while also elevating the risk of chronic diseases [41]. Elderly individuals suffering from insomnia tend to exhibit heightened concern for their sleep health and overall well-being, recognising the significance of health management in enhancing sleep quality and preventing complications. Consequently, they are more inclined to actively pursue health management services, such as sleep interventions and health monitoring. Additionally, a higher monthly income serves as a critical indicator of socioeconomic status [42].

Economic resources establish a material foundation that enables the elderly to access paid health services and reduces the economic barriers to health management. Concurrently, high-income groups typically enjoy superior living conditions and possess greater health awareness, which leads them to invest more in health and to be willing to pay for personalised and standardised health management services. This behaviour significantly elevates their demand for health management [43]. Elderly individuals with higher educational attainment generally exhibit enhanced health literacy, a stronger capacity to anticipate health risks, and a greater awareness of self-management. Consequently, they are more inclined to replace ineffective self-treatment with standardised and systematic health management services, thereby increasing their likelihood of seeking health management [44, 45]. A survey conducted in Portugal indicates a robust correlation between occupational status and health literacy [46]. Elderly individuals engaged in formal or informal regular occupations typically benefit from more comprehensive social security and medical resources throughout their working lives, fostering a strong understanding and habitual use of health services. Furthermore, this demographic tends to possess higher professional qualifications and cognitive abilities, facilitating their acceptance of contemporary health management concepts. They also have more accessible channels for obtaining health services, resulting in a significantly greater likelihood of health management demand compared to those in non-formal occupations, which aligns with the findings of He Beili and Zhang Yutong et al. [47, 48].

This study suggests that elderly individuals who infrequently undergo medical check-ups are more likely to have unmet health management needs. The prolonged absence of check-ups may have caused a build-up of undisclosed health risks and delayed risk awareness, leading this demographic to accumulate a higher number of unresolved health management gaps. Consequently, their health management requirements are elevated. Elderly individuals with limited self-care capabilities encounter challenges in sustaining their daily health due to declining physiological functions. They heavily depend on external professional assistance to compensate for their limitations and avert the risk of disability, thus exhibiting a heightened demand for health management. The findings reveal that elderly individuals with a high school education, those earning between 1500 and 3000 yuan per month, those who do not undergo regular medical check-ups, and those earning between 800 and 1500 yuan with a high school education have increased health management needs. This trend may stem from the beneficial combined impact of educational attainment, financial circumstances, and health-related behaviours.Elderly individuals with a high school education, who exhibit specific cognitive abilities and fall within the middle-income bracket of “monthly income 1500–3000 yuan” or “800–1500 yuan”, and who demonstrate the health behaviour of “not undergoing medical check-ups”, are more likely to recognise their own health risks and resource limitations. This awareness consequently fosters a heightened need for health management to compensate for deficiencies in health behaviours and to address potential health issues.

This study employed independent samples t-tests to analyze health management needs among older adults based on individual factors. Results indicate that older adults who understand chronic disease risk factors, actively learn about health management, use smartphones or other smart devices, measure health indicators at home, maintain a positive mindset, and express satisfaction with their current life circumstances are more likely to have health management needs.Elderly individuals who understand chronic disease-related factors can clearly recognize disease progression patterns and health risks, leading to more explicit demands for the prevention and management of chronic disease. Consequently, they exhibit a stronger willingness to actively seek professional health management services. This aligns with Xie Wanhua’s findings [49]. Older adults who proactively learn health management knowledge continuously enhance their health literacy, accurately identify their health vulnerabilities, and understand the scientific value of health management. Their proactive approach to health maintenance translates into actual demand for professional health management services, similar to findings from relevant studies in the United States and China [50, 51]. Elderly individuals using smart devices can conveniently access health information and connect with online health services, lowering barriers to obtaining health management information and services. Simultaneously, the health-monitoring functions of these devices further focus their attention on personal health, thereby increasing demand for health management. This aligns with the findings of studies by Xie H and Tang, LF, among others [52, 53]. Elderly individuals who measure health indicators at home gain real-time awareness of their health dynamics through measurement results. They can promptly detect abnormal indicators and perceive health risks, increasing the likelihood that they have health management needs. This aligns with the findings of Gao Yue et al. [54]. Elderly individuals with a positive mindset exhibit stronger self-efficacy and higher aspirations for quality of life and health. This enhances their motivation to modify health behaviors, thereby increasing their willingness to seek health management services [55]. Elderly individuals who are satisfied with their current living conditions are more likely to preserve their quality of life. Health is the core element that sustains the quality of life, leading people to proactively adopt health management to prevent disease and maintain wellness. Consequently, the probability of meeting their health management needs increases significantly [56].

This study employed multiple-choice items to assess the current state of health management in rural communities and gather suggestions from older adults regarding community-based health management models. See Table 6 for details. Findings indicate that health management coverage and service provision in Guangxi’s rural communities exhibit uneven distribution. Only slightly less than half of older adults have established personalized health records (49.14%), and a minority have participated in free health education activities (30.58%), reflecting room for improvement in both the precision and accessibility of community health management. Meanwhile, 41.65% of communities prioritize the spiritual well-being of the elderly, indicating that rural communities in ethnic regions are beginning to address the psychological health needs of the elderly. However, systematic service provision has yet to be established.

Regarding channels for acquiring disease knowledge, 58.66% of the elderly rely on television and radio, 51.33% obtain information through friends and family, and only 31.05% utilize community-based disease education resources. This aligns with findings from a Vietnamese study [57]. This disparity reveals the non-professional nature of health information dissemination in rural communities, where traditional media and interpersonal communication remain primary channels. The community’s role as a provider of specialized health knowledge has yet to be fully realized. Limited access to information among rural elderly may hinder improvements in their health literacy and the transformation of their health needs [58].

Regarding health management improvement needs, 80.66% of seniors expect “regular health monitoring and screening,” 69.42% demand “enhanced health management education,” and 68.8% seek “targeted chronic disease management services.” The overlap among these three high-demand areas aligns with findings by Di Jing et al. [59]. This reflects a shift in the health management needs of rural community seniors from passive treatment toward an “active prevention-continuous management” model.

This study demonstrates a deficiency in community service provision, exemplified by the establishment rate of health records, which stands at a mere 49.14%. On one hand, it is imperative to augment the capacity for professional health information supply within the community and to broaden dissemination channels that align with the characteristics of rural communities in underdeveloped cities in China. Particularly in ethnic regions, leveraging the “network of acquaintances” is essential for integrating modern health management concepts into the traditional culture of mutual assistance, thereby mitigating cultural resistance to service promotion.On the other hand, leveraging the predictive model developed in this study, resources such as personalized health records and regular monitoring should be prioritized for elderly populations with high health management needs. This approach will achieve precise matching of health management services and enhance their effectiveness.

### Strengths and limitations of the study

This study’s strengths lie in three dimensions: the uniqueness of its research setting and subject selection, the innovation of its methodology, and the practical relevance of its conclusions. First, the study focuses on rural communities in Guangxi, a region characterized by underdevelopment and distinct ethnic regional features. The selected research sample reflects both the commonalities of underdeveloped rural areas in western China and the specificities of health management for elderly populations in ethnic minority-populated areas, such as the Zhuang region, thereby enhancing the regional representativeness and specialized research value of the findings. Second, the study combines Lasso regression integrated with Logistic regression for variable selection, comparative validation of three machine learning algorithms, and SHAP interpretability analysis. This approach overcomes the limitations of traditional single regression analysis in health management demand research. It not only identifies the optimal predictive tool through the Random Forest model but also quantifies the mechanisms of core influencing factors using SHAP values.

Additionally, it supplements demand drivers at the individual level using independent-samples t-tests, thereby enhancing the scientific rigor of the methodology and the credibility of the results. Third, the findings align with practical needs in grassroots health management within rural communities. By identifying characteristics and recommendations for elderly populations with high health management demands, the study provides a reference basis for relevant departments to formulate tiered, categorized health management strategies tailored to underdeveloped ethnic regions in China and to optimize the allocation of rural community health service resources, demonstrating substantial practical application value.

However, this study has limitations: First, the cross-sectional design only reveals associations between variables, failing to clarify the causal sequence and the dynamic evolution of the relationship between health management needs and influencing factors. Longitudinal follow-up studies are needed to further validate causal mechanisms. Second, the sample originates solely from rural communities in Guangxi, reflecting characteristics of underdeveloped ethnic regions but failing to account for geographical variations across other underdeveloped rural areas in China. This study revealed that the influence of ethnic identity was primarily manifested indirectly through the variable of socioeconomic status (SES). However, this may have obscured the distinct impact of specific ethnic cultural traditions on health behaviours. Furthermore, as this research predominantly employed non-probability sampling methods, specifically stratified multi-stage convenience sampling, the representativeness of the sample was constrained. Consequently, when generalising these findings to other cultural contexts, ethnic compositions, or regions with varying levels of economic development, it is imperative to exercise caution and avoid overgeneralisation.Future research should expand to multi-regional, multi-ethnic cross-sample studies to broaden the applicability of these findings.

Additionally, the model incorporates demographic, economic, and health-behavior variables. However, it has not fully accounted for the regulatory role of macro factors, such as policies governing the allocation of primary medical resource, high-level regulatory variables, like “funding standards for national basic public health service projects”, “subsidy policies for family doctor contracting services”, and “pilot policies for long-term care insurance”, can directly impact individuals at the micro-level, particularly low-income individuals.Furthermore, the predictive accuracy of the model on the validation set has decreased in comparison to the training set, suggesting that enhancements are required to improve the model’s capacity to handle sample diversity. Future improvements to the model’s performance may involve the incorporation of multi-level models and the augmentation of macro-policy variables.Moreover, the assessment of social functions and children’s relationships in this study employs solely the Likert five-point scale, which may compromise the validity and reliability of the measurements. Additionally, the evaluation of health management needs primarily relies on the subjective reports of the elderly and utilises a single binary variable for definition. This approach obscures the heterogeneity and multi-faceted nature of health management needs and may introduce bias due to subjective cognitive errors. Future research should eschew single indicators in favour of multi-dimensional scales or discrete choice experiments (DCE) that have been validated for reliability and validity. It is essential to conduct a thorough quantitative assessment of health management needs across multiple dimensions, including type, intensity, and willingness to pay. Employing other established indicator scales will enhance the rigor of measurements and the comparability of results. Concurrently, integrating objective data, such as service records and health archives from primary healthcare institutions, will facilitate a more comprehensive and accurate evaluation of the health management needs of the elderly population.

## Conclusion

In conclusion, this study, which utilized machine learning algorithms to construct a predictive model, predicted the health management needs of the elderly in rural communities in China’s underdeveloped regions, revealing the characteristics of the needs of permanent elderly residents in rural communities in Guangxi and similar underdeveloped regions in western China. Among them, the XGB model demonstrated high accuracy, stability, and reliability. The SHAP analysis indicated that the five key characteristics influencing health management needs were health activities, per capita monthly income, regular physical examinations, education level, and occupation. Although there were slight differences in feature rankings among different models, this reflects the multi-dimensional importance of each factor under different algorithm logics, and the core driving factor set remains highly consistent. The interpretable predictive model created based on SHAP has significant practical application value and can serve as empirical reference materials for relevant departments in regions with similar social and economic backgrounds and population structures to effectively assess the health management needs of the elderly and provide personalized interventions. Future research can design larger-scale prospective randomized controlled trials and include macro-policy variables and cultural-specific indicators to further verify the applicability of the XGB model in practical applications.

## Supplementary Information

Supplementary Material 1: Fig A1 OBB error convergence plot and error variation rate analysis for elderly health management needs predicted by the RF model. Table B1 Descriptive Statistics of Variables for Individuals Aged 60 and Above in Guangxi (*N*=641). Table B2 Multivariate logistic regression analysis of health management needs among older adults. Table B3 Variable Assignments for Elderly Health Management Needs.

## Data Availability

The datasets used and/or analysed during the current study are available from the corresponding author on reasonable request.

## Abbreviations

HMN: Health management needs
PCR: Parent-child relationship
LR: Logistic Regression
RF: Random Forest
XGB: Extreme Gradient Boosting
