# Supplementary material for: Unraveling the mechanisms of health management needs among rural elderly in underdeveloped Chinese regions: a machine learning approach to predictive model building and factor analysis
Source: BMC Geriatr. 2026 Mar 28;26:786. doi: 10.1186/s12877-026-07368-z (PMC13227739; doi:10.1186/s12877-026-07368-z)
Supplement: Supplementary file 1 — Supplementary Material 1: Fig A1 OBB error convergence plot and error variation rate analysis for elderly health management needs predicted by the RF model. Table B1 Descriptive Statistics of Variables for Individuals Aged 60 and Above in Guangxi (N=641). Table B2 Multivariate logistic regression analysis of health management needs among older adults. Table B3 Variable Assignments for Elderly Health Management Needs. [file 12877_2026_7368_MOESM1_ESM.docx]

1. **supplementary figure**


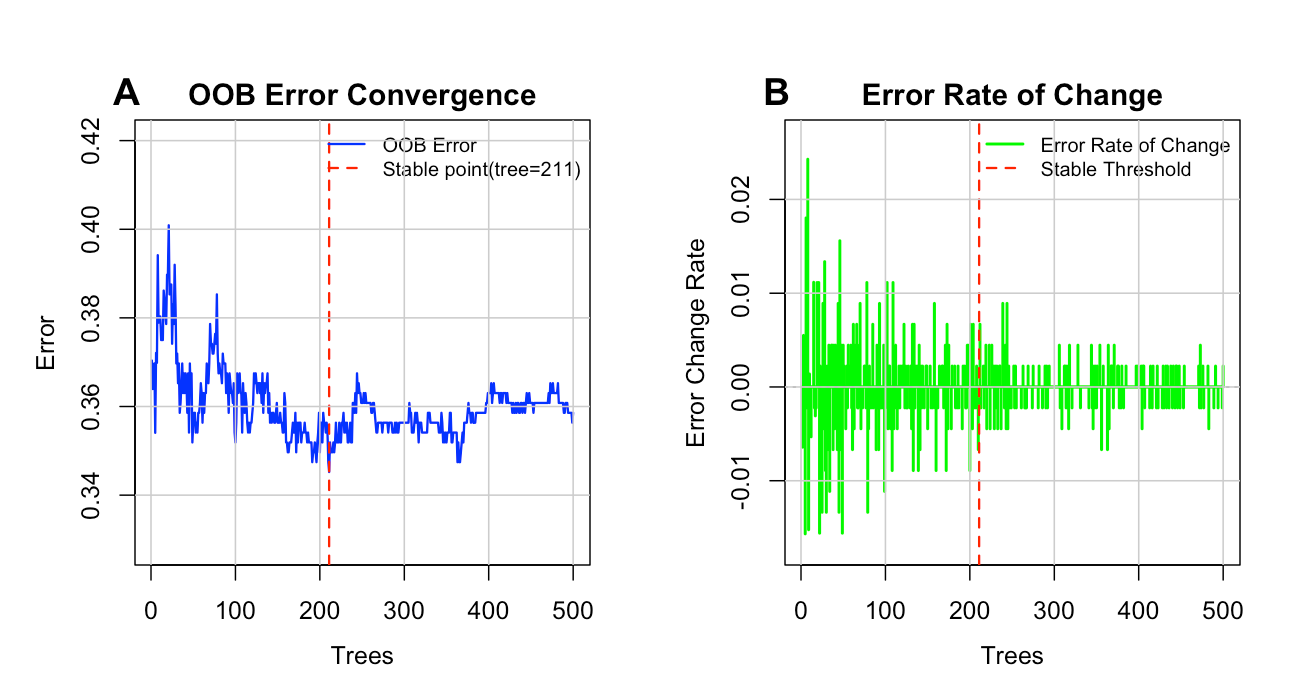


**Fig A1** OBB error convergence plot and error variation rate analysis for elderly health management needs predicted by the RF model

1. **supplementary tables**

**B1-B3**

**Table B1** Descriptive Statistics of Variables for Individuals Aged 60 and Above in Guangxi (N=641)

| **Variables** | **Total   (n=641)** | **Non-HMN(n=360)** | **HMN (n=281)** | **X^2^** | **P** |
| --- | --- | --- | --- | --- | --- |
| Household Registration Status |  |  |  | 12.523 | <0.001 |
| Rural | 448 (69.9) | 272 (75.6) | 176 (62.6) |  |  |
| Urban | 193 (30.1) | 88 (24.4) | 105 (37.4) |  |  |
| Age (years) |  |  |  | 12.499 | <0.01 |
| 60～ | 152 (23.7) | 74 (20.6) | 78 (27.8) |  |  |
| 65～ | 189 (29.5) | 97 (26.9) | 92(32.7) |  |  |
| 70～ | 155 (24.2) | 103 (28.6) | 52 (18.5) |  |  |
| ≥75 | 145 (22.6) | 86 (23.9) | 59 (21.0) |  |  |
| Spouse |  |  |  | 1.748 | 0.186 |
| No | 186 (29.0) | 112 (31.1) | 74 (26.3) |  |  |
| Yes | 455 (71.0) | 248 (68.9) | 207 (73.7) |  |  |
| Educational level |  |  |  | 23.308 | <0.001 |
| Elementary school and below | 316 (49.3) | 203 (56.4) | 113 (40.2) |  |  |
| Junior high/vocational school | 143 (22.3) | 79 (21.9) | 64 (22.8) |  |  |
| High School | 93 (14.5) | 39 (10.8) | 54 (19.2) |  |  |
| College/Undergraduate | 62 (9.7) | 30 (8.3) | 32 (11.4) |  |  |
| Bachelor's degree or above | 27 (4.2) | 9 (2.5) | 18 (6.4) |  |  |
| Occupation |  |  |  | 27.647 | <0.001 |
| Party and government organs/public institutions personnel | 97 (15.1) | 45 (12.5) | 52 (18.5) |  |  |
| Enterprise employees | 58 (9.0) | 24 (6.7) | 34 (12.1) |  |  |
| Farmers | 306 (47.7) | 178 (49.4) | 128 (45.6) |  |  |
| Self-employed | 67 (10.5) | 35 (9.7) | 32 (11.4) |  |  |
| Freelancing | 60 (9.4) | 33 (9.2) | 27 (9.6) |  |  |
| Unemployed | 53 (8.3) | 45(12.5) | 8(2.8) |  |  |
| Monthly per capita income (RMB) |  |  |  | 25.482 | <0.001 |
| ＜800 | 108 (16.8) | 80 (22.2) | 28 (10.0) |  |  |
| 800～ | 194(30.3) | 118 (32.8) | 76 (27.0) |  |  |
| 1500～ | 234 (36.5) | 111 (30.8) | 123 (43.8) |  |  |
| ≥3000 | 105 (16.4) | 51 (14.2) | 54 (19.2) |  |  |
| Health Insurance Type |  |  |  | 6.363 | 0.095 |
| Urban Employee Medical Insurance | 87 (13.6) | 39 (10.8) | 48 (17.1) |  |  |
| Urban and Rural Residents' Medical Insurance | 517 (80.7) | 298 (82.8) | 219 (77.9) |  |  |
| Commercial Insurance | 10 (1.6) | 5 (1.4) | 5 (1.8) |  |  |
| None | 27(4.2) | 18 (5.0) | 9(3.2) |  |  |
| Duration of chronic disease (years) |  |  |  | 3.004 | 0.699 |
| None | 213 (33.2) | 113 (31.4) | 100 (35.6) |  |  |
| 0～ | 100(15.6) | 54 (15.0) | 46(16.4) |  |  |
| 3～ | 128 (20.0) | 71(19.7) | 57(20.3) |  |  |
| 5～ | 135(21.1) | 82(22.8) | 53(18.9) |  |  |
| 10～ | 40 (6.2) | 25(6.9) | 15(5.3) |  |  |
| ≥15 | 25 (3.9) | 15 (4.2) | 10 (3.6) |  |  |
| History of critical illness |  |  |  | 0.216 | 0.642 |
| No | 545 (85.0) | 304 (84.4) | 241 (85.8) |  |  |
| Yes | 96 (15.0) | 56 (15.6) | 40 (14.2) |  |  |
| Health Check Frequency |  |  |  | 28.276 | <0.001 |
| Every six months | 72 (11.2) | 28 (7.8) | 44 (15.7) |  |  |
| Once a year | 201 (31.4) | 93 (25.8) | 108 (38.4) |  |  |
| Irregular | 292 (45.6) | 189 (52.5) | 103 (36.7) |  |  |
| Rarely | 76 (11.9) | 50 (13.9) | 26 (9.3%) |  |  |
| Smoking |  |  |  | 1.027 | 0.598 |
| No | 381 (59.4) | 219 (60.8) | 162 (57.7) |  |  |
| Quit | 128 (20.0) | 67 (18.6) | 61 (21.7) |  |  |
| Yes | 132 (20.6) | 74(20.6) | 58 (20.6) |  |  |
| Alcohol consumption |  |  |  | 3.592 | 0.166 |
| No | 346 (54.0) | 201 (55.8) | 145 (51.6) |  |  |
| Quit | 116 (18.1) | 56 (15.6) | 60 (21.4) |  |  |
| Yes | 179 (27.9) | 103 (28.6) | 76 (27.0) |  |  |
| Insomnia status |  |  |  | 0.419 | 0.518 |
| No | 390 (60.8) | 223 (61.9) | 167 (59.4) |  |  |
| Yes | 251 (39.2) | 137 (38.1) | 114 (40.6) |  |  |
| Health Activities |  |  |  | 50.179 | <0.001 |
| Frequently | 67 (10.5) | 21 (5.8) | 45 (16.4) |  |  |
| Occasionally | 188 (29.3) | 97 (26.9) | 91 (32.4) |  |  |
| Rarely | 176 (27.5) | 86 (23.9) | 90 (32.0) |  |  |
| Never | 210 (32.8) | 156 (43.3) | 54 (19.2) |  |  |
| Self-care ability |  |  |  | 0.552 | 0.759 |
| Able to care for oneself | 526 (82.1) | 297 (82.5) | 229 (81.5) |  |  |
| Requires appropriate assistance from others | 102 (15.9) | 57 (15.8) | 45 (16.0) |  |  |
| Unable to care for oneself | 13 (2.0) | 6 (1.7) | 7 (2.5) |  |  |
| Children's Emotional Support |  |  |  | 15.380 | <0.01 |
| Very Good | 157 (24.5) | 70 (19.4) | 87 (31.0) |  |  |
| Good | 314 (49.0) | 180 (50.0) | 134 (47.7) |  |  |
| Average | 132 (20.6) | 86 (23.9) | 46 (16.4) |  |  |
| Not very good | 22 (3.4) | 12 (3.3) | 10 (3.6) |  |  |
| Poor | 16(2.5) | 12 (3.3) | 4(1.4) |  |  |
| Social Functioning |  |  |  | 28.797 | <0.001 |
| Very Good | 111 (17.3) | 42 (11.7) | 69 (24.6) |  |  |
| Good | 284(44.3) | 154 (42.8) | 130 (46.3) |  |  |
| Average | 196 (30.6) | 133 (36.9) | 63 (22.4) |  |  |
| Not very good | 40 (6.2) | 23 (6.4) | 17 (6.0) |  |  |
| Poor | 10 (1.6) | 8 (2.2) | 2 (0.7) |  |  |

**Table B2** Multivariate logistic regression analysis of health management needs among older adults

| Variable | Estimate | Std.Error | Wald | Pr(>\|z\|) | OR | OR 95CI |
| --- | --- | --- | --- | --- | --- | --- |
| (Intercept) | -0.601 | 0.758 | 0.628 | 0.428 | 0.548 | 0.124-2.443 |
| Household registration2 | 0.17 | 0.286 | 0.352 | 0.553 | 1.185 | 0.674-2.077 |
| Age2 | -0.062 | 0.304 | 0.042 | 0.838 | 0.94 | 0.517-1.706 |
| Age3 | -0.503 | 0.329 | 2.342 | 0.126 | 0.605 | 0.316-1.148 |
| Age4 | -0.208 | 0.347 | 0.357 | 0.55 | 0.813 | 0.41-1.604 |
| Education2 | 0.182 | 0.304 | 0.36 | 0.548 | 1.2 | 0.66-2.175 |
| Education3 | 0.457 | 0.35 | 1.703 | 0.192 | 1.579 | 0.796-3.148 |
| Education4 | 0.196 | 0.444 | 0.194 | 0.659 | 1.216 | 0.504-2.898 |
| Education5 | 1.102 | 0.62 | 3.157 | 0.076 | 3.01 | 0.924-10.765 |
| Occupation2 | 0.489 | 0.474 | 1.064 | 0.302 | 1.631 | 0.648-4.182 |
| Occupation3 | 0.259 | 0.406 | 0.406 | 0.524 | 1.295 | 0.586-2.892 |
| Occupation4 | 0.135 | 0.5 | 0.073 | 0.787 | 1.145 | 0.429-3.07 |
| Occupation5 | -0.24 | 0.496 | 0.235 | 0.628 | 0.786 | 0.295-2.075 |
| Occupation6 | -0.88 | 0.601 | 2.145 | 0.143 | 0.415 | 0.122-1.305 |
| Spouse1 | 0.312 | 0.267 | 1.37 | 0.242 | 1.366 | 0.812-2.316 |
| Monthly income2 | 0.662 | 0.384 | 2.959 | 0.085 | 1.938 | 0.924-4.196 |
| Monthly income3 | 1.027 | 0.384 | 7.156 | 0.008 | 2.794 | 1.336-6.052 |
| Monthly income4 | 0.763 | 0.455 | 2.808 | 0.094 | 2.144 | 0.885-5.302 |
| Health insurance2 | 0.087 | 0.41 | 0.045 | 0.832 | 1.091 | 0.49-2.463 |
| Health insurance3 | 0.187 | 0.89 | 0.044 | 0.834 | 1.206 | 0.205-7.147 |
| Health insurance4 | 0.814 | 0.709 | 1.316 | 0.251 | 2.256 | 0.547-9.034 |
| Smoke2 | 0.088 | 0.279 | 0.1 | 0.752 | 1.092 | 0.63-1.888 |
| Smoke3 | 0.25 | 0.29 | 0.739 | 0.39 | 1.284 | 0.726-2.271 |
| Insomnia1 | 0.387 | 0.231 | 2.799 | 0.094 | 1.472 | 0.937-2.324 |
| Check up2 | -0.333 | 0.398 | 0.702 | 0.402 | 0.717 | 0.325-1.554 |
| Check up3 | -0.734 | 0.398 | 3.412 | 0.065 | 0.48 | 0.218-1.041 |
| Check up4 | -0.464 | 0.491 | 0.895 | 0.344 | 0.629 | 0.238-1.638 |
| Self care2 | 0.442 | 0.31 | 2.032 | 0.154 | 1.556 | 0.848-2.868 |
| Self care3 | 0.305 | 0.909 | 0.112 | 0.738 | 1.356 | 0.204-7.86 |
| Health activities2 | -0.127 | 0.424 | 0.09 | 0.764 | 0.88 | 0.379-2.014 |
| Health activities3 | 0.206 | 0.434 | 0.227 | 0.634 | 1.229 | 0.521-2.878 |
| Health activities4 | -1.052 | 0.457 | 5.312 | 0.021 | 0.349 | 0.141-0.849 |
| Social capability2 | -0.489 | 0.342 | 2.047 | 0.152 | 0.613 | 0.311-1.194 |
| Social capability3 | -0.903 | 0.413 | 4.785 | 0.029 | 0.405 | 0.178-0.905 |
| Social capability4 | -0.474 | 0.585 | 0.656 | 0.418 | 0.622 | 0.194-1.942 |
| Social capability5 | -0.875 | 0.984 | 0.79 | 0.374 | 0.417 | 0.047-2.58 |
| PCR2 | -0.055 | 0.292 | 0.035 | 0.851 | 0.947 | 0.534-1.684 |
| PCR3 | 0.246 | 0.398 | 0.381 | 0.537 | 1.278 | 0.587-2.801 |
| PCR4 | 0.891 | 0.736 | 1.464 | 0.226 | 2.437 | 0.564-10.381 |
| PCR5 | 0.783 | 0.891 | 0.772 | 0.38 | 2.188 | 0.344-12.218 |

**Table B3** Variable Assignments for Elderly Health Management Needs

| Variables | Assignment mode |
| --- | --- |
| Household registration | Rural=1; Urban=2 |
| Age | 60–64=1; 65–69=2; 70–74=3; ≥75=4 |
| Education | Primary school or below=1; Junior high/Technical secondary=2; High school=3; College/Bachelor=4; Postgraduate and above=5 |
| Occupation | Government/Institution staff=1; Enterprise employee=2; Farmer=3; Self-employed=4; Freelancer=5; Unemployed=6 |
| Spouse | No=0; Yes=1 |
| Monthly income | <800=1; 800–1499=2; 1500–2999=3; ≥3000=4 |
| Health insurance | Urban employee medical insurance=1; Urban-rural resident medical insurance=2; Commercial insurance=3; None=4 |
| Smoke | No=1; Former smoker=2; Current smoker=3 |
| Insomnia | No=0; Yes=1 |
| Check up | Every 6 months=1; Every year=2; Irregular=3; Almost never=4 |
| Self care | Fully independent=1; Needs assistance=2; Dependent=3 |
| Health activities | Regular participation=1; Occasional participation=2; Rare participation=3; No participation=4 |
| Social capability | Very good=1; Good=2; Fair=3; Poor=4; Very poor=5 |
| PCR | Very good=1; Good=2; Fair=3; Poor=4; Very poor=5 |
